# Using contextual and lexical features to restructure and validate the classification of biomedical concepts

**DOI:** 10.1186/1471-2105-8-264

**Published:** 2007-07-24

**Authors:** Jung-Wei Fan, Hua Xu, Carol Friedman

**Affiliations:** 1 Department of Biomedical Informatics, Columbia University Vanderbilt Clinic, 5th Floor, 622 West 168th Street, New York, NY 10032, USA

## Abstract

**Background:**

Biomedical ontologies are critical for integration of data from diverse sources and for use by knowledge-based biomedical applications, especially natural language processing as well as associated mining and reasoning systems. The effectiveness of these systems is heavily dependent on the quality of the ontological terms and their classifications. To assist in developing and maintaining the ontologies objectively, we propose automatic approaches to classify and/or validate their semantic categories. In previous work, we developed an approach using contextual syntactic features obtained from a large domain corpus to reclassify and validate concepts of the Unified Medical Language System (UMLS), a comprehensive resource of biomedical terminology. In this paper, we introduce another classification approach based on words of the concept strings and compare it to the contextual syntactic approach.

**Results:**

The string-based approach achieved an error rate of 0.143, with a mean reciprocal rank of 0.907. The context-based and string-based approaches were found to be complementary, and the error rate was reduced further by applying a linear combination of the two classifiers. The advantage of combining the two approaches was especially manifested on test data with sufficient contextual features, achieving the lowest error rate of 0.055 and a mean reciprocal rank of 0.969.

**Conclusion:**

The lexical features provide another semantic dimension in addition to syntactic contextual features that support the classification of ontological concepts. The classification errors of each dimension can be further reduced through appropriate combination of the complementary classifiers.

## Background

### Introduction

Biomedical ontologies such as Gene Ontology (GO) [1], the Foundational Model of Anatomy (FMA) [2], and the Unified Medical Language System (UMLS) [3,4] are important for terminology management, data sharing/integration, and decision support [5]. The ontologies specify not only the definitions of biomedical terms but also associate them with normalized concepts and semantic categories within the ontological structures. Therefore, they provide abundant lexical and semantic knowledge that is especially valuable to Natural Language Processing (NLP) systems. The overhead involved in the costly and time-consuming system development process could be substantially reduced with the aid of these knowledge sources. NLP techniques have been playing an increasingly critical role in bioinformatics research [6-8]. Spasic et al. [9] summarized various approaches that applied ontologies in biomedical NLP tasks.

High-quality semantic classification is crucial for NLP and for other ontology-based applications that take advantage of conceptualization and reasoning, because the semantic accuracy can affect the correctness and/or the flexibility of the applications. We have been investigating automated methods to assist in developing and maintaining the semantic classification of biomedical ontologies for NLP purposes. The goal is two-fold: to directly improve the ontologies and to indirectly improve the NLP applications built upon the ontologies. Our work currently focuses on semantic classification within the UMLS because it has evolved [10] to include vocabularies such as NCBI taxonomy [11], GO, and OMIM [12], making it valuable not only for clinical applications but for bioinformatics applications as well. In addition to currently being the most comprehensive resource of biomedical terms, the UMLS has a broad user population within the biomedical domain, and is continually maintained by the National Library of Medicine (NLM).

In a previous work [13] we demonstrated the feasibility of using a corpus-based, distributional similarity approach to semantic classification of UMLS concepts that appear in text. An evaluation of the method demonstrated that it achieved a lowest error rate of 0.198. The distributional approach was based on Harris' sublanguage theory stating that the syntactic dependence of words on other words exhibits unequal likelihood constraints especially in specialized domain [14]. For example, in the biomedical domain the head noun of the adjective "homologous" is more likely to be a *gene or protein *than a *disorder*, and such unequal distribution of syntactic dependences can be used to characterize different semantic categories. Subsequently, we recognized that in the biomedical domain the terms used to name the concepts are generally descriptive, and can be as important as the contextual information for characterizing semantic categories. For example, words such as "syndrome", "deficiency", and "malignant" occur frequently in concepts of the *disorder *class and thus should be statistically representative of the class. Therefore, in this paper we developed a string-based classification approach and compared it with the distributional approach. The string-based approach uses words constituting the concepts. We hypothesized that it is feasible to characterize a semantic class through the lexical usage of terms associated with concepts in that class.

Both the distributional and the string-based approaches are automated and are based on empirical language usage, so they differ from manually classifying the concepts and should perform more consistently and objectively than humans. We expect that the automated methods will assist human experts by proposing classifications in a high-throughput manner. Additionally, the semantic classes used by the two approaches can be defined with varying coverage or granularity, preserving the flexibility to customize the classifiers for different applications. To the best of our knowledge, this is the first time the string-based approach has been used and compared with a distributional approach for semantically classifying UMLS concepts.

In the following subsections we introduce background material concerning the methods and resources related to our study, as well as the issues involved, and then describe the experiments, followed by the results, discussion, conclusion, and details of the methods.

### The UMLS Semantic Network

The current release (2007AA) of UMLS integrates 139 source vocabularies into a concept-centered terminology, with each concept assigned a Concept Unique Identifier (CUI). Each CUI is assigned to one or more semantic types in the Semantic Network (SN) [15], which specifies semantic relations between the semantic types. The biomedical knowledge in the SN is preserved in both the semantic classification and the semantic relations. For example, type-relation-type triplets such as T195:("Antibiotic") **interrupts **T043:("Cell Function") encode propositions that can be used for various purposes. The UMLS concepts and their semantic types have been used by some NLP applications to determine relations among the extracted terms through designated semantic patterns [16,17]. Although semantic classification is crucial to the NLP applications, Cimino et al. have reported inconsistencies [18] in the SN classification. The SN is also known to associate numerous concepts with questionable semantic types. For example, the current release assigns "Cavitated nodule", "Calcified nodule", and "Ossified nodule" to T169 "Functional Concept", but "Functional Concept" is very general and these terms are semantically closer to T190 "Anatomical Abnormality".

Another issue involved in the SN classification is that the granularity may be appropriate for certain knowledge-based applications but may be too fine-grained for general biomedical NLP systems. For example, in the previous work we grouped "Fungus", "Virus", "Rickettsia or Chlamydia", "Bacterium", and "Archaeon" into a single *microorganism *class because, for that group, the textual patterns and textual relations with other biomedical entities are generally similar. From an ontological perspective, Burgun et al. [19] also suggested that the SN types should be further simplified to form basic-level semantic categories. There has been research on simplifying [20-22] and auditing [23-25] the UMLS semantic classification. However, the approaches relied on existing semantic types to form broader categories as well as audit questionable semantic assignments, so they could not avoid the limitations of the existing SN structure. For example, if a gene has been assigned to only T025 "Cell" from the very beginning, it will be still incorrectly grouped into an anatomy-related broad class without any contradictions detected. In other words, re-organizing the semantic types *in situ *does not help remove the erroneous semantic assignments, and none of the auditing methods provides automated classification(s) for individual concepts. Therefore, in this paper we propose approaches different from the above related work in that we reclassify the concepts directly and automatically.

### Corpus-based semantic similarity

The corpus-based approach derived from Harris' distributional hypothesis [26] states that terms can be semantically characterized and classified by the distribution of frequently co-occurring terms associated with specific syntactic relations. For example, a syntactic dependency of "cellular defense response" in the sample sentence "Cellular defense response is affected by temperature" would be **passive_verb**(affected), denoting that "cellular defense response" is the object of "affected". If we zoom in a little bit, we can also specify the syntactic dependency **modifier_adjective**(cellular) for the term "defense response". In general, the syntactic dependencies serve as more informative features for semantic classification, and it has been reported in related work [27,28] that they outperformed simpler approaches that used only co-occurring terms without considering syntax. From a parsed corpus, we can extract sets of such syntactic dependencies for individual concepts (e.g. "cellular defense response") and for semantic classes (e.g. *biologic function*). The syntactic dependencies of each concept are collected, assigned weights, and normalized into a probability distribution profile for the concept. In order to create a distributional profile for each class, a similar procedure is followed. In this case, the syntactic dependencies of all the concepts associated with the class are aggregated, assigned weights, and normalized to form its distributional profile. Through the distributional profiles, we can perform classification by computing the semantic similarities between the profile of an individual concept and that of a set of semantic classes. Similarity measures (e.g. Lee's α-skew divergence [29]) that quantify the overlapping between probability distributions can be used, and a concept will be classified as the semantic class with which it has the highest similarity score. Readers may refer to our previous work [13] for more details about the methods.

In the biomedical domain, Sibanda et al. [30] aimed to show that syntactic dependencies are useful for determining the semantic categories of terms and clauses in discharge summaries, though a Support Vector Machine was used instead of a canonical distributional model. They defined eight semantic categories, including complex categories such as *results*. Some of the categories can be defined directly as a subset of SN types, but some (e.g. *dosages*) have no SN type equivalents. Using syntactic features along with orthographic and lexical features, *F*-measures of >90% were achieved for most categories. Weeds et al. [31] applied their co-occurrence retrieval distributional similarity method based on syntactic dependencies and a nearest neighbor voting process to classify terms into the 36 semantic categories of the GENIA corpus [32], achieving a best accuracy of 0.768. In our previous work [13] of reclassifying UMLS concepts into seven broad semantic categories, we used syntactic dependencies that were initially extracted from shallow-parsed PubMed abstracts and then organized into concept-based distributional profiles for each of the categories. Lee's α-skew divergence was used as the similarity metric, and a best accuracy of 0.802 was achieved. For this paper we applied the same method to build our distributional classifiers but built them using a larger training corpus and incorporated some minor implementation refinements.

### String-based approach to semantic similarity

Lexical features (e.g. content words or phrases) are usually used in text categorization tasks, with wide applications in general NLP such as news topic classification [33] and authorship verification [34] (see Sebastiani's review [35] on text categorization). Regardless of the text size, tasks dealing with small pieces of text can still be considered as a special case of text categorization. For example, once a topic classifier is built, it can be used to classify headlines consisting of even only a few words. In the biomedical domain, words as well as other processed lexical tokens were shown to be useful for named entity recognition/classification. For example, Lee et al. [36] built SVM classifiers based on both contextual (surrounding words and bigrams) and lexical features to classify terms into GENIA semantic categories. Working also on GENIA semantic classification, Torii et al. [37] applied a decision list algorithm and found lexical features more helpful than contextual features (adjacent words). In Zhang and colleague's work of partitioning the Semantic Network [22], words of the SN type definitions were used to compute semantic similarity for grouping the types.

The Naïve Bayesian model [38] is widely used by text categorization systems. In Naïve Bayesian classification the target posterior probability for a specific class *c*_i _given a set of features *F *is computed as:

P(*c*_i_|F) = P(*F*|*c*_i_)·P(*c*_i_)/P(*F*)

≈P(*f*_1_, *f*_2_, ..., *f*_n_|*c*_i_)·P(*c*_i_)     (omit the common denominator)

≈P(*f*_1_|*c*_i_)·P(*f*_2_|*c*_i_)·...·P(*f*_n_|*c*_i_)·P(*c*_i_)     (assume conditional independence)

The correct class is predicted as the *c*_i _which maximizes the product of formula **3**. For this paper we classify UMLS concepts into high-level semantic categories such as *biologic function *and *disorder*, i.e. the *c*_i _in the above formulas. The features by our approach are words of the strings associated with each CUI. For example, the string "cellular defense response" of the concept C1155076 provides "cellular", "defense", and "response" as the features of the concept. The prior probabilities P(*c*_i_) and the conditional probabilities P(*f*_j_|*c*_i_) can both be estimated from the training data. By pooling the words of multiple CUIs under certain SN type or arbitrarily defined high-level semantic class, a larger lexical profile can be formed for Naïve Bayesian classification. The details of the implementation are described in Methods.

### The UMLS for building the string-based classifier

The UMLS has well-defined SN types and some less well-defined, semantically general or vague SN types. SN types such as T047 "Disease or Syndrome" are well-defined, and the concepts under them are semantically homogeneous and could reliably be used for training, whereas some types are not appropriate for building classifiers. For example, T033 "Finding", is very general. It includes concepts corresponding to biological functions such as "Basal gastric acid output" and "Nitrogen balance", but also includes many disorder-related concepts such as "Hyperlactatemia", "Progressive renal failure", and some very general findings such as "Unemployment" and "Birth place". By pooling lexical features of well-defined SN types into semantic classes we can use the UMLS to train the Naïve Bayesian model introduced earlier, while excluding those less well-defined SN types during the training process.

A subset of the UMLS source vocabularies is known for well-established ontological structure. In addition, some have semantic annotations embedded in the UMLS strings. For example, some GO terms contain a parenthesized "sensu" note for specifying taxonomical information, e.g. "cell wall polysaccharide anabolism (sensu Fungi)". Similar but not strictly ontological, the HUGO Nomenclature [39] has some taxonomical notes parenthesized as in "mindbomb homolog 2 (Drosophila)" or some semantic qualifiers parenthesized as in "endothelial cell growth factor 1 (platelet-derived)". Cohen and colleagues have reported that parenthesized information contributes to naming variants within and between genes [40]. Another valuable source ontology is SNOMED-CT [41] because it has sound ontological properties and a broad coverage of biomedicine. SNOMED semantic classes such as "Disease", "Body structure", "Function", and "Substance" occasionally occur in the UMLS string terms as parenthesized annotations, e.g. "Oxidative phosphorylation (function)". These parenthesized annotations provide extra information of the ontological views from different source vocabularies. Therefore, in building the Naïve Bayesian classifiers we experimented with features consisting of only the pure strings (i.e. discarding parenthesized annotations) and those consisting of strings including the annotations. To reduce training noise, we also excluded strings that were marked as suppressible synonyms because they were considered obsolescent by the corresponding source vocabularies or by the NLM.

### Summary of experiments in the current study

We evaluated the string-based approach and compared it with the distributional approach for classifying UMLS concepts into eight broad semantic classes: *anatomy *(above the molecular level), *behavior*, *biologic function*, *disorder*, *gene or protein*, *microorganism*, *procedure*, and *substance*. We chose these specific classes because according to the recent reviews of the field [6-8] they comprised the most relevant ones for biomedical text mining applications. For each approach, the eight classes were trained using 64 well-defined SN types ' [see Additional file 1]' out of the total 135, while noisy types such as T033 discussed in the previous section were not used. For the distributional approach we varied the number of available syntactic dependencies that were used, and for the string-based approach we used strings either with or without parenthesized annotations. The two approaches were compared on the same test sets. The gold standard was automatically generated based on the CUIs that had their SN types modified within the 2005AA~ 2006AA interval (by comparing the UMLS MRSTY.RRF tables). The gold standard had been evaluated by a biomedical expert with an M.D. degree using a subset of CUIs that were randomly sampled from the testing data. The expert's agreement with the gold standard's classification was more than 86% (43/50), and therefore the gold standard was determined to be reliable. For methodological details concerning the gold standard creation and evaluation, please refer to our previous work [13].

The main quantitative evaluation reported in this paper consists of the error rates of the different automated classifiers. Each error rate was computed by the formula:

M+0.5⋅TN
 MathType@MTEF@5@5@+=feaafiart1ev1aaatCvAUfKttLearuWrP9MDH5MBPbIqV92AaeXatLxBI9gBaebbnrfifHhDYfgasaacH8akY=wiFfYdH8Gipec8Eeeu0xXdbba9frFj0=OqFfea0dXdd9vqai=hGuQ8kuc9pgc9s8qqaq=dirpe0xb9q8qiLsFr0=vr0=vr0dc8meaabaqaciaacaGaaeqabaqabeGadaaakeaadaWcaaqaaiabd2eanjabgUcaRiabicdaWiabc6caUiabiwda1iabgwSixlabdsfaubqaaiabd6eaobaaaaa@362B@

where *M *is the number of misclassifications, *T *is the number of ties, and *N *is the total number of CUIs tested. A tie is defined when the correct or incorrect predictions receive equal similarity scores from the classifier. The correct/incorrect classification counting for a single test CUI was divided by the number of classes it was associated with in the gold standard. For example, if in the gold standard a CUI belonged to both *gene or protein *and *substance*, and in the top 2 predictions the classifier only got *substance*, it was counted as a 0.5 correct classification and a 0.5 misclassification. The error rate can be understood as a measure complementary to classification accuracy, with tied cases penalized as a half misclassification. We also applied linear combinations varying the relative weights of the two approaches and evaluated the error rates of the hybrid classifiers.

To evaluate the efficacy of the classifiers' ranking function (i.e. the ability of bringing the correct class up to the topmost prediction), we calculated the mean reciprocal rank (MRR) for each experiment:

∑(1/Rt)T
 MathType@MTEF@5@5@+=feaafiart1ev1aaatCvAUfKttLearuWrP9MDH5MBPbIqV92AaeXatLxBI9gBaebbnrfifHhDYfgasaacH8akY=wiFfYdH8Gipec8Eeeu0xXdbba9frFj0=OqFfea0dXdd9vqai=hGuQ8kuc9pgc9s8qqaq=dirpe0xb9q8qiLsFr0=vr0=vr0dc8meaabaqaciaacaGaaeqabaqabeGadaaakeaadaWcaaqaamaaqaeabaWaaeWaaeaacqaIXaqmcqGGVaWlcqqGsbGucqWG0baDaiaawIcacaGLPaaaaSqabeqaniabggHiLdaakeaacqWGubavaaaaaa@3606@

where *T *is the total number of targets to be predicted and R*t *is the rank of a specific target *t *among the classifier's predictions. MRR has been used in the TREC Genomics Track [42] for an information retrieval task similar to question-answering. To evaluate the ranking performance of our classifiers we adapted the MRR by assuming that the gold standard class was the target to be retrieved. The MRR value lies in the range of (0, 1), and a value of 1 is considered the best the performance.

## Results

### Error rates of individual approaches

The error rates computed by formula **4 **are presented in Table 1, where *N *represents the number of CUIs tested. The columns show results on test sets with varying number of syntactic dependencies (the complete test set disregarding syntactic dependencies, ≥ 1, and ≥ 10). The table also shows the MRR values of the individual experiments. The majority of the MRR values were above 0.85 (except for the distributional approach experiment where there were insufficient features), indicating that the ranking mechanism of the classifiers functioned well. The string-based approach achieved a highest MRR of 0.907 (when including parenthesized annotations), and the distributional approach achieved that of 0.887.

**Table 1:** Error rates of the experiments

	**The entire test set ** (*N *= 223)		**≥ 1 syntactic dependencies ** (*N *= 192)		**≥ 10 syntactic dependencies ** (*N *= 91)	
**Type of features**	Error rate	MRR	Error rate	MRR	Error rate	MRR

Syntactic dependencies	--	--	0.315	0.792	0.187	**0.887**
Strings without annotations*	0.191	0.881	0.221^#^	0.862^#^	0.247^#^	0.853^#^
Strings with annotations*	0.143	**0.907**	0.164^#^	0.895^#^	0.192^#^	0.886^#^

* Note that the string-based approach is independent of the number of available syntactic features used by the distributional approach, and the alignment (^#^) is just for comparison purpose.

The advantage of the string-based approach over the distributional approach was stronger on test sets where the latter obtained fewer features (e.g. the error rate 0.164 versus the 0.315 on the test CUIs with ≥ 1 syntactic dependencies). However, it should be clarified that the string-based approach is independent of the number of available syntactic features used by the distributional approach, and the alignment (the entires marked with ^#^ in the table) is just to allow for comparison on the same test sets. Therefore, the first column (entire test set) actually presents a general evaluation of the string-based approach, and the 223 test CUIs form the superset of all the other test sets in the table. It can also be estimated that about 86% (192/223) of the test CUIs had ≥ 1 syntactic dependencies for the eight selected broad classes, suggesting the proportion which the distributional approach influenced.

### Summary of the misclassifications

We manually analyzed the misclassifications in the column with ≥ 10 syntactic dependencies in Table 1. The distributional classifiers made 17 misclassifications (see Table 2 for the confusion matrix), of which the top 3 misclassified classes were: *biologic function *(6), *disorder *(3), and *procedure *(3). For example, "Down-regulation" was misclassified as *procedure*, and "Acetylation" was misclassified as *gene or protein*. The string-based classifier without using parenthesized annotations made 22.5 misclassifications, of which the top 3 misclassified classes were: *anatomy *(7), *substance *(6), and *biologic function *(5). For example, "Shoulder" and "Foot" were misclassified as *disorder*. The *substance *"Alkylating agents" was misclassified as *disorder*, and the *biologic function *"Anergy" was misclassified as *procedure*. There were 7 overlapping misclassifications between the 17 and 22.5, but 4 of them were misclassified as different incorrect classes.

**Table 2:** Confusion matrix for the 17 misclassifications by the distributional approach on the *N *= 91 test set

	1)	2)	3)	4)	5)	6)	7)	8)
1) anatomy			2					
2) behavior								
3) biologic function	1			2	2		1	
4) disorder			3					
5) gene or protein								
6) microorganism								
7) procedure		1	1	1				
8) substance				1		1		

The string-based classifier using parenthesized annotations made 17.5 misclassifications (see Table 3 for the confusion matrix), of which the top 3 misclassified classes were also: *anatomy *(5), *substance *(4), and *biologic function *(4), but each had fewer misclassifications as noted in parentheses. For example, the anatomical modifiers "Peritoneal" and "Popliteal" were both misclassified as *procedure*. The *substance *"Quinolones" was misclassified as *disorder*, and the *biologic function *"Hemolysis" was misclassified as *behavior*. By including parenthesized annotations, 6 of the 22.5 misclassifications by the pure string-based approach were corrected. However, the *procedure *"Acoustic Evoked Brain Stem Potentials" was misclassified as *biologic function *only after including the parenthesized annotations. Please ' [see Additional file 2, 3, 4]' for complete lists of the misclassifications in the three experiments summarized above.

**Table 3:** Confusion matrix for the 17.5 misclassifications by the string-based approach (with parenthesized annotations) on the *N *= 91 test set

	1)	2)	3)	4)	5)	6)	7)	8)
1) anatomy				1			3	1
2) behavior			1					
3) biologic function		3		1				
4) disorder		1	1					
5) gene or protein								1
6) microorganism								
7) procedure			1					
8) substance				3.5				

### Complementariness study

The three experiments concerning misclassification analysis summarized in the preceding section used the same test set (91 CUIs), and represented three approaches respectively: distributional, string-based, and string-based plus parenthesized annotations. We computed how many misclassifications by one approach were correct using the other approach. In Table 4 the values in each cell represent the proportion of how many misclassifications (denominator) by the approach represented by the row were correctly classified (numerator) by the approach represented by the column. For example, the top right value 12/17 means 12 out of 17 misclassifications by the distributional approach were correctly classified by the string-based approach with parenthesized annotations.

**Table 4:** Misclassifications that are complemented to be correct by different approaches

**Features used** **+ Similarity measure**	Distributional dependencies + α-skew divergence	Strings without annotations + Naïve Bayesian	Strings with annotations + Naïve Bayesian
Distributional dependencies + α-skew divergence	--	10/17	12/17
Strings without annotations + Naïve Bayesian	15.5/22.5	--	6/22.5
Strings with annotations + Naïve Bayesian	12.5/17.5	1/17.5	--

The first row indicates that more than half (10/17) of the misclassifications by the distributional approach were correct when using the string-based approach. The string-based approach was strengthened by the parenthesized annotations, recovering over two thirds (12/17) of the misclassifications by the distributional approach. The second row shows that more than two thirds (15.5/22.5) of the misclassifications by the pure string-based approach were correct using the distributional approach. The third row shows that more than two thirds (12.5/17.5) of the misclassifications by the string-based approach including annotations were correct using the distributional approach.

### Error rates of the combined approaches

Table 5 shows the error rates for linearly combined classifiers. The coefficient *w *in the table denotes the manually optimized weight for the distributional classifier, while 1-*w *was applied to weigh the string-based classifier complementarily. The lowest error rate of 0.055 was obtained by combining the distributional approach with ≥ 10 syntactic dependencies and the string-based approach that included parenthesized annotations. The advantage of combining was apparent especially when the distributional classifier was built with more contextual features because the error rate decreased from 0.143 to 0.055 as the number of dependencies increased from ≥ 1 to ≥ 10. The increase in MRR values shows that the ranking function also benefited from the hybrid effect. Implementation details of the linear combination are described in the Methods section.

**Table 5:** Error rates of the combined classifier

	**The entire test set ** (*N *= 223)		**≥ 1 syntactic dependencies ** (*N *= 192)		**≥ 10 syntactic dependencies ** (*N *= 91)	
**Type of features**	Error rate	MRR	Error rate	MRR	Error rate	MRR

Syntactic dependencies + Strings without annotations	0.191 *w *= 0	0.881	0.167 *w *= 0.2	0.900	0.110 *w *= 0.3	0.935
Syntactic dependencies + Strings with annotations	0.143 *w *= 0	0.907	0.143 *w *= 0.3	0.912	0.055 *w *= 0.3	0.969

* Note that in the first column we set *w *= 0 because the distributional approach is not applicable.

## Discussion

In this paper we classify ontological concepts for purposes of curation or customization for specific types of applications, whereas the related work [36,37] dealt with classification of terms occurring in text. One implication was that they could exploit positional cues for lexical features (e.g. head nouns usually reside around the rightmost) but we were prohibited by the prevalent permutation of ontological strings (e.g. "transcription, RNA-dependent") which rarely appear in real text. Therefore, we assumed a simpler order-insensitive, independent bag-of-words model. In Torii et al. [37], informative suffixes were also included as lexical features. For contextual features, we incorporated syntactic relations, while the related work used only surrounding terms. Our classifiers were built using the resources publicly offered by the NLM, and our semantic classes included higher-level entities than the specific ones of molecular biology tested in the related work. Our eight semantic classes covered the major entities the bioinformatics community is interested in. For example, *gene or protein *and *biologic function *are apparently relevant, while cellular components are covered under *anatomy*, phenotypes are covered by *behavior *and *disorder*, and *substance *covers chemicals and drugs. Please ' [see Additional file 1]' for detailed contents of the eight classes.

The results demonstrated that the performance was very good, especially when combining the two complementary approaches. The methods proposed in this paper can help validate classification of the CUIs used in training (e.g. the CUIs of the 64 SN types used in training our eight classes) and help reclassify CUIs in noisy but potentially useful SN types (e.g. T033 "Finding" and T169 "Functional Concepts"). New concepts added to an existing source vocabulary or that in a newly added source vocabulary can also be classified with the assistance of our methods. However, there are concepts of some SN types that are not specifically useful for biomedical applications (at least 30 of the 135), and we do not plan to reclassify them (e.g. "Urban Plannings" in T064 "Governmental or Regulatory Activity", "Democracy" in T078 "Idea or Concept", and "Algorithms" in T170 "Intellectual Product"). The methods are generalizable to any ontology wherever strings of the concepts are available and when the concepts can be identified in a training corpus. The training classes can be formed based on an existing ontological structure or by any arbitrary selection criteria, depending on the intended application. In the following subsections we discuss issues related to our results and methods.

### Analysis of the string-based approach

When using the string-based approaches, many *anatomy *concepts were misclassified as *disorder*, for example, C0016504 "Foot" and C0037004 "Shoulder". We observed that the words of these basic body parts also appear frequently in the strings of *disorder *concepts involving these body parts, e.g. "Mycetoma of foot" and "Congenital valgus deformity of foot". Similarly, two *anatomy *modifier adjectives "peritoneal" and "popliteal" were misclassified as *procedure *owing to the presence of numerous procedure terms like "Continuous ambulatory peritoneal dialysis" and "Incision of popliteal space". However, some concepts had overlapping classes, and could be considered correctly classified as both classes. For example, C0431085 "Unspecified tumor cell NOS" is semantically a *disorder *and an *anatomy *(as an abnormal type of cell). Several *substance *concepts were misclassified as *disorder*. We found the unique term "Macrolides" of C0282563 appear frequently in concepts such as "poisoning by macrolides" or "macrolides causing adverse effects in therapeutic use", which belong to the SN type "Injury or Poisoning" (selected in training our *disorder *class). As a result, the representative string of the concept prevails more in the *disorder *class rather than in the *substance *class. The same situation happened to C0034428 "Quinolones" and C0002073 "Alkylating agents", e.g. the latter has strings frequently involved in a type of therapy-related acute myeloid leukaemia (*disorder*).

Overall, the string-based approach was an unexpected success, since it is based on a very simplified compositional view concerning meaning: concepts can be semantically characterized by their lexical components (e.g. "single simple ovarian cyst") regardless of the order and syntactic roles of the components. Possibly this is due to characteristics relating to the descriptive way in which biomedical concepts are named, which basically corresponds to noun phrases, in which case the syntactic roles within the noun phrases may not be as important as syntactic roles within complete sentences. For example, in "cellular defense response", the fact that "cellular" and "defense" are modifiers of "response" is similar to the fact that they are words comprising the term. The results demonstrated that the simple approach did not compromise performance much while it avoided execution of a more complex and difficult task involving syntactic analysis. In the error analysis, however, we observed a typical disturbance to the Naïve Bayesian model: features (words) that are not semantically representative of certain classes occur frequently in those classes because of compositionality. For example, many words of the *anatomy *class occur frequently in *disorder *or *procedure *concepts, as discussed earlier. This implied that for test CUIs with only simple strings (and usually consisting of single words), the conditional probabilities that had been overestimated in certain classes might adversely affect the classification. If a test CUI consists of only a few non-discriminative words (with close conditional probabilities over every class), the classification could also be determined more by the priors of the classes. We anticipate the problem would still exist when applied to a more specific ontology such as GO, in which prevalent compositional structures have been reported by Ogren et al [43]. For example, many substance terms such as "abscisic acid" are embedded in both Biological Process (e.g. "abscisic acid mediated signaling") and Molecular Function (e.g. "abscisic acid binding activity") concepts, but the presence of "abscisic acid" does not help much in determining which of the classes the concept should be assigned. A possible solution would be to perform syntactic analysis in order to determine the head nouns (e.g. the "signaling" and "activity" above) and to assign them higher weights when building the string-based classifier. However, this is left for future work.

The result that string-based models trained with parenthesized annotations performed the best implies that many of the annotations from the source vocabularies agree with the SN types, and therefore the annotations provided straightforward support in making the correct classification. For example, by the augmented string-based approach "Foot" and "Shoulder" were correctly classified owing to the class annotation *body structure *from SNOMED-CT and *anatomy *from the Psychological Index Terms. Although these annotations are useful for obtaining the best reclassification, they could be based on manual curation and thus are not completely objective and are prone to human inconsistencies. However, if the manual re-assignment process is independent of the source classification, the parenthesized annotations could be thought as the surrogates of certain high-level intensional (i.e. to philosophically define a concept by stating its properties) meanings from different source vocabularies and in the string-based approach they contributed a type of highly weighted feature. Along with the strings, the annotations (whenever available) were aggregated under each CUI to serve as an extensional (i.e. to philosophically define a concept by enumerating its instances) meaning of the concept, as interpreted by Campbell et al. [44]. Therefore, when the annotations of more than one or two source vocabularies consistently favor a semantic class incompatible to the SN type, we would doubt the appropriateness of the SN type assignment. To summarize, although the annotations are useful, further study is required to determine how objective they are.

### Issues in comparing and combining the two approaches

In Table 4 we have shown how the two approaches are quantitatively complementary. Although the string-based approach seemed generally more robust, the distributional approach with sufficient features could make a decisive contribution to the combined model. For example, our results showed that about 41% (91/223) of the test CUIs could be classified with an error rate of 0.055 (see Table 5). If the training corpus for the distributional approach were made larger, it is likely that the error rate would be even lower. The confusion matrices also revealed qualitative complementariness of the two approaches in terms of their relative strength on different classes. For example, a cluster of *anatomy *concepts misclassified as *procedure *(with two examples discussed earlier) were only observed in the string-based approach. This sheds light on solutions of applying class-dependent combination for the two approaches in the future, and we will need larger testing data to plot richer and more stable confusion matrices for analysis.

Since the distributional approach uses contextual features and different similarity metrics, it is less susceptible to the weakness of the Naïve Bayesian model. An interesting example was C0066928 "Mullerian-inhibiting hormone", assigned to *substance *in the gold standard. The string-based approaches misclassified it as *biologic function*, probably because it contained the substring "inhibiting" while the distributional approach correctly classified it as *gene or protein *(top 1) and *substance *(top 2). We verified that it is a member of the transforming growth factor-beta gene family (e.g. in *Homo sapiens*, Entrez GeneID: 268) by searching the NCBI database. This also demonstrates that the approach could capture a more specific sense to augment the existing SN classification. However, we observed that the distributional approach incurred different types of misclassifications. The typical misclassifications by the distributional approach resulted from inadequate discriminating power of the syntactic dependencies. For example, the molecular function C0001038 "Acetylation" was misclassified as *gene or protein*, indicating that the function was not differentiated from the molecule itself through the contextual information.

The string-based approach we used was based on CUIs and the strings associated with the CUIs. Many of the UMLS concepts are complex multi-word phrases, which are compositional. In such a phrase the meaning is based on a combination of the meanings of the parts constituting the phrase (e.g. "ribosomal protein S6 kinase") as a single concept. When using the distributional approach, however, the entire phrase representing a concept is treated as an atomic entity and therefore the modifiers within that phrase (e.g. "ribosomal") are not counted as syntactic dependencies. As a result, when using the distributional approach based on concepts many syntactic dependencies were lost that would have been obtained if we were using just words. In contrast, when using the string-based approach the loss of contextual features associated with multi-word concept terms are inadvertently compensated for because the words of the strings are utilized in building the classifier. For example, the word "ribosomal", which is characteristic of the *gene or protein *class, actually occurred 583 times P(ribosomal|*gene or protein*) = 0.00158 in the string-based profile but only 33 times P(**modifier_adjective**(ribosomal)|*gene or protein*) = 0.00018) as a modifier adjective in the distributional profile, demonstrating in this case, that the distributional information was more strongly captured by the string-based model than by the distributional model. This phenomenon suggests why the string-based approach performed better than the distributional approach because when informative adjuncts, such as "ribosomal", were absorbed into the UMLS concepts, the syntactic dependencies of some *gene or protein *concepts might have become indistinguishable from that of the more general *substance *class.

There were also cases where the two approaches agreed with each other, but disagreed with the gold standard. It seemed that some of them represent borderline categories where the concepts could be semantically interpreted in more than one way. For example, C0079319 "Acoustic Evoked Brain Stem Potentials", which according to the UMLS definition means electrical waves generated in the brain when responding to auditory click stimuli. Therefore this concept refers to the functional condition of the brain stem as determined by a diagnostic procedure. It was classified by the distributional approach as *biologic function*, but the gold standard class was *procedure*. The string-based approach using parenthesize annotations agreed with the distributional approach, since the concept was annotated as *function *in SNOMED-CT. The examples discussed above also indicate that the classification accuracy might be underestimated a little by using the automatically generated gold standard.

### Limitations

It is possible that the SN updates from the UMLS, which we used to automatically obtain the gold standard, are not completely objective for testing because they might represent more difficult cases that had been misclassified even by human curators in earlier releases. In addition, they may not be completely error-free. Although we believe that the semantic classes selected in this study have covered the major entities that the bioinformatics community is interested in, the constituent SN types included/excluded based on our own judgment according to recent reviews [6-8] may not be free of bias. The performance of the distributional approach in this paper was bound to the randomly sampled training corpus, so that both the optimal size and representativeness could not be guaranteed.

### Future work

We plan to 1) use syntactic analysis of the phrasal structures to improve the feature weighting of the string-based approach, 2) seek other gold standard sources that would offer greater randomness and testing size, 3) develop an automatic method to determine how to optimally combine the two approaches, 4) use the hybrid classifier to reclassify potentially useful concepts under general and vague SN types that were not used for training, e.g. T033 "Finding", 5) compare our results with other machine learning algorithms based on the same existing features, 6) propose a revised classification scheme for the UMLS, and 7) integrate the new classification into NLP applications.

## Conclusion

We developed a string-based approach to classify UMLS concepts into eight broad semantic classes, and compared its performance with that of a method based on a distributional approach, which we previously developed. The performance of the string-based approach appeared to be comparable to the distributional approach, with an error rate of 0.143 and a mean reciprocal rank of 0.907. By comparing misclassifications associated with the distributional and string-based approaches, the two were found to be complementary. Classification performance was further enhanced after applying linear combinations of the two approaches, achieving the lowest error rate of 0.055 and a mean reciprocal rank of 0.969. Based on our results, we conclude that it is highly feasible to use the hybrid classifier to assist human experts in validating/reclassifying UMLS concepts.

## Methods

### Determining the training classes and their CUIs

**Figure 1:**
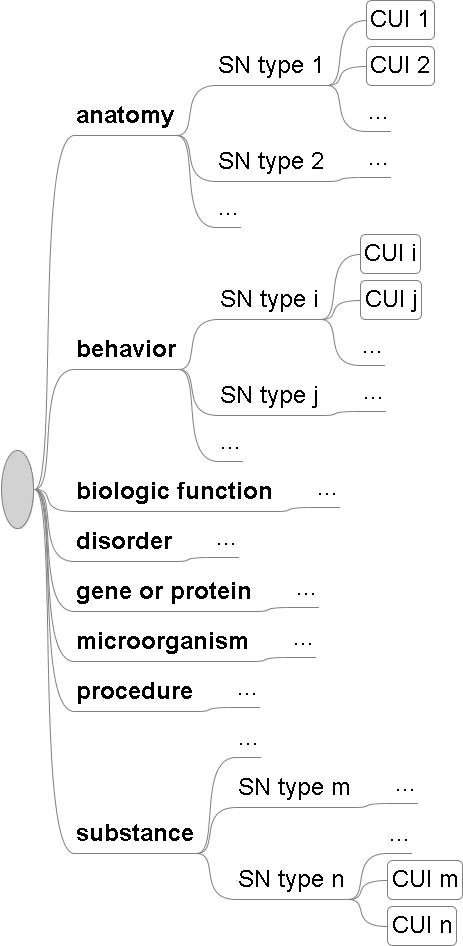
Hierarchical illustration of the eight semantic classes (with some of the constituent SN types and CUIs omitted for conciseness).

In the previous work, we formed seven broad semantic classes that we considered to be most significant for biomedical applications. In this paper, we added the *behavior *class in order to cover concepts related to behavioral phenotypes (e.g. C0242659 "Female homosexuality). We then selected 64 well-defined (out of a total of 135) SN types and mapped them into eight broad classes to make each of them semantically coherent. For example, the *biologic function *class included T038 "Biologic Function", T039 "Physiologic Function", T040 "Organism Function", T042 "Organ or Tissue Function", T043 "Cell Function", T044 "Molecular Function", and T045 "Genetic Function". Please ' [see Additional file 1]' for the complete list of SN types under each class. The CUIs belonging to each class were automatically identified, so that each broad class contained multiple SN types and included all their underlying CUIs (as illustrated by Figure 1). All the CUIs in each class were then used to train the classifier, as elaborated below.

### Training the string-based Naïve Bayesian classifier

The Naïve Bayesian classifier was built based on words in the UMLS concept strings. Suppressible strings that have been obsolesced (flagged as 'O') by the source or by the NLM were excluded, e.g. string variations simply with an additional note *NOS *(Not Otherwise Specified) were excluded in this process. From the 2006AA MRCONSO.RRF table, we selected the unique English strings of each CUI. By unique strings, we mean the strings corresponding to the non-redundant String Unique Identifier (SUI) pertaining to each CUI. The selected strings were broken into words and transformed to lowercase. All punctuations were removed, but lexical variants (e.g. inflections or derivational suffixes) were not normalized for simplicity of implementation. Words consisting of only one character (e.g. "1" or "i") or those belonging to the PubMed stoplist [45] were discarded; these words were considered non-informative for semantic classification and constituted about 0.05% of all the distinct words extracted. For example, from the string texts of Table 6 we extracted the individual words and their frequencies as displayed in Table 7. Then via the string words from all the CUIs associated with each class, we calculated the prior probabilities of the classes and the conditional probabilities of the words given each class (for use in formula **3 **to train the classifier). Two versions of the classifier were trained, using strings either with or without parenthesized annotations. For words not seen in the trained models, their prior probabilities were used for smoothing.

**Table 6:** Example strings of the concept C0079380

**String Unique Identifier**	**String text**
S6441909	Frameshift Mutation function
S0042786	Frameshift Mutation
S6148830	Reading Frame Shift Mutation
S6364685	Out-of-Frame Mutation

**Table 7:** Words and frequencies obtained from the string texts of Table 6

**Word**	**Frequency**
frameshift	2
mutation	4
function	1
reading	1
frame	2
shift	1
out	1

### Evaluating the two classification approaches

Based on our previous method of automatically generating test CUIs and the corresponding gold standard, we obtained 223 CUIs that belonged to the eight broad classes and were unambiguously identified from the training corpus (199,313 processed PubMed abstracts) used in our previous work. The 223 CUIs with gold standard classification served as the testing data and had been excluded from training the classifiers. We did not consider CUIs that never occurred in the corpus since there are many CUIs that would never be found in text, and our objective in semantic classification is primarily to improve text mining tasks. Using only the CUIs that occur in biomedical text is practical for these tasks, as McCray et al. [46] have reported that only about 10% of the UMLS strings actually occur in MEDLINE. The string-based approach was evaluated on the entire test set of 223 CUIs. Then we varied the number of available contextual features (≥ 1 and ≥ 10 syntactic dependencies) for the distributional approach and obtained two other subsets for testing (192 and 91 respectively out of the entire set of 223), and the two approaches were both evaluated on the two subsets for comparison. The gold standard was derived according to the 2006AA SN classification, and based on that the error rate (formula **4**) and mean reciprocal rank (formula **5**) were computed. We manually analyzed and summarized misclassifications by each approach on the test set (91 CUIs) where the distributional approach had ≥ 10 features. Confusion matrices for the misclassifications were also generated. To evaluate whether the two approaches were complementary, a program was created to automatically compute the number of misclassifications by one approach that were correctly classified when using the other.

### Linear combination of the two approaches

Before applying a linear combination, the raw similarity scores of each approach were rescaled and normalized into pseudo-probabilities by the following formulas respectively:

[log(min(*X*))/log(*x*)] - 1     (for Natïve Bayesian)

[max(*X*)/*x*] - 1     (for α-skew divergence)

where *X *represents the similarity scores between the test CUI and all the eight classes and *x *is the similarity score of the specific class being considered. Then the following formula was applied to combine the adjusted scores of the two approaches:

*w*·SD + (1 - *w*)·NB

where SD and NB stand respectively for the adjusted scores of the distributional and the string-based approach, and *w *was varied from 0.1 to 0.9 with an interval of 0.1. The combined similarity scores for each CUI were computed so that a score was obtained for each of the eight semantic classes, and then the eight scores were sorted in descending order, so that the class with the highest combined score was taken the top prediction.

## Authors' contributions

JF developed the programs, conducted the experiments, and prepared the manuscript. JF and HX both conducted the data analysis. The research was performed under the advice and supervision of CF. All authors read and approved the final manuscript.

## Supplementary Material

Additional file 1 The 8 broad semantic classes and their constituent SN types. A detailed list of the Semantic Types selected to build each of our eight broad classes. Click here for file

Additional file 2 Summary of the 17 misclassifications by the distributional approach. A more detailed list of the misclassified concepts by the distributional approach. Click here for file

Additional file 3 Summary of the 22.5 misclassifications by the string-based approach without parenthesized annotations. A more detailed list of the misclassified concepts by the string-based approach without using the parenthesized annotations. Click here for file

Additional file 4 Summary of the 17.5 misclassifications by the string-based approach with parenthesized annotations. A more detailed list of the misclassified concepts by the string-based approach using the parenthesized annotations. Click here for file

## References

[B1] Ashburner M, Ball CA, Blake JA, Botstein D, Butler H, Cherry JM, Davis AP, Dolinski K, Dwight SS, Eppig JT (2000). Gene ontology: tool for the unification of biology. The Gene Ontology Consortium. Nat Genet.

[B2] Rosse C, Mejino JL (2003). A reference ontology for biomedical informatics: the Foundational Model of Anatomy. J Biomed Inform.

[B3] Lindberg DA, Humphreys BL, McCray AT (1993). The Unified Medical Language System. Methods Inf Med.

[B4] Campbell KE, Oliver DE, Shortliffe EH (1998). The Unified Medical Language System: toward a collaborative approach for solving terminologic problems. J Am Med Inform Assoc.

[B5] Yu AC (2006). Methods in biomedical ontology. J Biomed Inform.

[B6] Cohen AM, Hersh WR (2005). A survey of current work in biomedical text mining. Brief Bioinform.

[B7] Natarajan J, Berrar D, Hack CJ, Dubitzky W (2005). Knowledge discovery in biology and biotechnology texts: a review of techniques, evaluation strategies, and applications. Crit Rev Biotechnol.

[B8] Jensen LJ, Saric J, Bork P (2006). Literature mining for the biologist: from information retrieval to biological discovery. Nat Rev Genet.

[B9] Spasic I, Ananiadou S, McNaught J, Kumar A (2005). Text mining and ontologies in biomedicine: making sense of raw text. Brief Bioinform.

[B10] Bodenreider O (2004). The Unified Medical Language System (UMLS): integrating biomedical terminology. Nucleic Acids Res.

[B11] The NCBI Entrez Taxonomy. http://www.ncbi.nlm.nih.gov/entrez/query.fcgi?db=Taxonomy.

[B12] Online Mendelian Inheritance in Man, OMIM (TM). http://www.ncbi.nlm.nih.gov/sites/entrez?db=OMIM.

[B13] Fan JW, Friedman C (2007). Semantic classification of biomedical concepts using distributional similarity. J Am Med Inform Assoc.

[B14] Harris ZS (1991). A theory of language and information: a mathematical approach.

[B15] McCray AT (2003). An upper level ontology for the biomedical domain. Comp Funct Genom.

[B16] Rindflesch TC, Libbus B, Hristovski D, Aronson AR, Kilicoglu H (2003). Semantic relations asserting the etiology of genetic diseases. AMIA Annu Symp Proc.

[B17] Rindflesch TC, Fiszman M (2003). The interaction of domain knowledge and linguistic structure in natural language processing: interpreting hypernymic propositions in biomedical text. J Biomed Inform.

[B18] Cimino JJ, Min H, Perl Y (2003). Consistency across the hierarchies of the UMLS Semantic Network and Metathesaurus. J Biomed Inform.

[B19] Burgun A, Botti G, Fieschi M, Le Beux P (1999). Sharing knowledge in medicine: semantic and ontologic facets of medical concepts. IEEE Proc Syst Mans Cybern.

[B20] McCray AT, Burgun A, Bodenreider O (2001). Aggregating UMLS semantic types for reducing conceptual complexity. Medinfo.

[B21] Chen Z, Perl Y, Halper M, Geller J, Gu H (2002). Partitioning the UMLS semantic network. IEEE Trans Inf Technol Biomed.

[B22] Zhang L, Perl Y, Halper M, Geller J, Hripcsak G (2005). A lexical metaschema for the UMLS semantic network. Artif Intell Med.

[B23] Gu H, Perl Y, Elhanan G, Min H, Zhang L, Peng Y (2004). Auditing concept categorizations in the UMLS. Artif Intell Med.

[B24] Cimino JJ (1998). Auditing the Unified Medical Language System with semantic methods. J Am Med Inform Assoc.

[B25] Peng Y, Halper MH, Perl Y, Geller J (2002). Auditing the UMLS for redundant classifications. Proc AMIA Symp.

[B26] Harris ZS (1968). Mathematical structures of language.

[B27] Cimiano P, Völker J (2005). Towards large-scale, open-domain and ontology-based named entity classification. Proc Intl Conf Recent Adv Nat Lang Process.

[B28] Hatzivassiloglou V, Klavans JL, Resnik P (1996). Do we need linguistics when we have statistics? A comparative analysis of the contributions of linguistic cues to a statistical word grouping system. The balancing act: combining symbolic and statistical approaches to language.

[B29] Lee L (1999). Measures of distributional similarity. Proc Annu Meet Assoc Comput Linguist.

[B30] Sibanda T, He T, Szolovits P, Uzuner O (2006). Syntactically-informed semantic category recognition in discharge summaries. AMIA Annu Symp Proc.

[B31] Weeds J, Dowdall J, Schneider G, Keller B, Weir D (2005). Using distributional similarity to organize biomedical terminology. Terminology.

[B32] Kim JD, Ohta T, Tateisi Y, Tsujii J (2003). GENIA corpus – semantically annotated corpus for bio-textmining. Bioinformatics.

[B33] Calvo RA, Lee J, Li X (2004). Managing content with automatic document classification. J Digit Inf.

[B34] Diederich J, Kindermann O, Leopold E, Paass G (2003). Authorship attribution with support vector machines. APPL INTELL.

[B35] Sebastiani F, Zanasi A (2005). Text categorization. Text mining and its applications.

[B36] Lee KJ, Hwang YS, Kim S, Rim HC (2004). Biomedical named entity recognition using two-phase model based on SVMs. J Biomed Inform.

[B37] Torii M, Kamboj S, Vijay-Shanker K (2004). Using name-internal and contextual features to classify biological terms. J Biomed Inform.

[B38] Mitchell TM (1997). Machine Learning.

[B39] Wain HM, Lush MJ, Ducluzeau F, Khodiyar VK, Povey S (2004). Genew: the Human Gene Nomenclature Database, 2004 updates. Nucleic Acids Res.

[B40] Cohen KB, Acquaah-Mensah GK, Dolbey AE, Hunter L Contrast and variability in gene names. Proceedings of Workshop on NLP in the Biomedical Domain, ACL 2002; Philadelphia.

[B41] SNOMED: SNOMED CT. http://www.ihtsdo.org/our-standards/snomed-ct/.

[B42] TREC Genomics Track – Roadmap. http://ir.ohsu.edu/genomics/roadmap.html.

[B43] Ogren PV, Cohen KB, Acquaah-Mensah GK, Eberlein J, Hunter L (2004). The compositional structure of Gene Ontology terms. Pac Symp Biocomput.

[B44] Campbell KE, Oliver DE, Spackman KA, Shortliffe EH (1998). Representing thoughts, words, and things in the UMLS. J Am Med Inform Assoc.

[B45] PubMed stopwords. http://www.ncbi.nlm.nih.gov/books/bv.fcgi?rid=helppubmed.table.pubmedhelp.T43.

[B46] McCray AT, Bodenreider O, Malley JD, Browne AC (2001). Evaluating UMLS strings for natural language processing. Proc AMIA Symp.

