# Supplementary material for: Using contextual and lexical features to restructure and validate the classification of biomedical concepts
Source: BMC Bioinformatics. 2007 Jul 24;8:264. doi: 10.1186/1471-2105-8-264 (PMC2014782; doi:10.1186/1471-2105-8-264)
Supplement: Additional file 1 — The 8 broad semantic classes and their constituent SN types. A detailed list of the Semantic Types selected to build each of our eight broad classes. [file 1471-2105-8-264-S1.pdf]

## The 8 broad semantic classes and their constituent SN types

(columns under each class: SN type code, SN type abbreviation, SN type name)

**Group: anatomy**

| | | |
|---|---|---|
| T017 | anst | Anatomical_Structure |
| T018 | emst | Embryonic_Structure |
| T021 | ffas | Fully_Formed_Anatomical_Structure |
| T022 | bdsy | Body_System |
| T023 | bpoc | Body_Part_Organ_or_Organ_Component |
| T024 | tisu | Tissue |
| T029 | blor | Body_Location_or_Region |
| T030 | bsoj | Body_Space_or_Junction |
| T025 | cell | Cell |
| T026 | celc | Cell_Component |

**Group: microorganism**

| | | |
|---|---|---|
| T004 | fngs | Fungus |
| T005 | virs | Virus |
| T006 | rich | Rickettsia_or_Chlamydia |
| T007 | bact | Bacterium |
| T194 | arch | Archaeon |

**Group: disorder**

| | | |
|---|---|---|
| T019 | cgab | Congenital_Abnormality |
| T020 | acab | Acquired_Abnormality |
| T037 | inpo | Injury_or_Poisoning |
| T047 | dsyn | Disease_or_Syndrome |
| T048 | mobd | Mental_or_Behavioral_Dysfunction |
| T049 | comd | Cell_or_Molecular_Dysfunction |
| T184 | sosy | Sign_or_Symptom |
| T190 | anab | Anatomical_Abnormality |
| T191 | neop | Neoplastic_Process |

**Group: gene_or_protein**

| | | |
|---|---|---|
| T028 | gngm | Gene_or_Genome |
| T086 | nusq | Nucleotide_Sequence |
| T087 | amas | Amino_Acid_Sequence |
| T114 | nnon | Nucleic_Acid_Nucleoside_or_Nucleotide |
| T116 | aapp | Amino_Acid_Peptide_or_Protein |
| T192 | rcpt | Receptor |

**Group: biologic_function**

| | | |
|---|---|---|
| T038 | biof | Biologic_Function |
| T039 | phsf | Physiologic_Function |
| T040 | orgf | Organism_Function |
| T042 | ortf | Organ_or_Tissue_Function |

| | | |
|---|---|---|
| T043 | celf | Cell_Function |
| T044 | moft | Molecular_Function |
| T045 | genf | Genetic_Function |

**Group: procedure**

| | | |
|---|---|---|
| T059 | lbpr | Laboratory_Procedure |
| T060 | diap | Diagnostic_Procedure |
| T061 | topp | Therapeutic_or_Preventive_Procedure |

**Group: behavior**

| | | |
|---|---|---|
| T053 | bhvr | Behavior |
| T054 | socb | Social_Behavior |
| T055 | inbe | Individual_Behavior |

**Group: substance**

| | | |
|---|---|---|
| T121 | phsu | Pharmacologic_Substance |
| T127 | vita | Vitamin |
| T195 | antb | Antibiotic |
| T111 | eico | Eicosanoid |
| T115 | opco | Organophosphorus_Compound |
| T118 | carb | Carbohydrate |
| T119 | lipd | Lipid |
| T123 | bacs | Biologically_Active_Substance |
| T124 | nsba | Neuroreactive_Substance_or_Biogenic_Amine |
| T130 | irda | Indicator_Reagent_or_Diagnostic_Aid |
| T131 | hops | Hazardous_or_Poisonous_Substance |
| T196 | elii | Element_Ion_or_Isotope |
| T197 | inch | Inorganic_Chemical |
| T125 | horm | Hormone |
| T110 | strd | Steroid |
| T103 | chem | Chemical |
| T104 | chvs | Chemical_Viewed_Structurally |
| T109 | orch | Organic_Chemical |
| T120 | chvf | Chemical_Viewed_Functionally |
| T126 | enzy | Enzyme |
| T200 | clnd | Clinical_Drug |
