# Supplementary material for: Using contextual and lexical features to restructure and validate the classification of biomedical concepts
Source: BMC Bioinformatics. 2007 Jul 24;8:264. doi: 10.1186/1471-2105-8-264 (PMC2014782; doi:10.1186/1471-2105-8-264)
Supplement: Additional file 2 — Summary of the 17 misclassifications by the distributional approach. A more detailed list of the misclassified concepts by the distributional approach. [file 1471-2105-8-264-S2.pdf]

Summary of the 17 misclassifications by the distributional approach

CUI	Concept	Predicted	Gold Standard (GS) class & SN types	GS class ranking*
C0037004	Shoulder	biologic_function	anatomy (T023 Body Part, Organ, or Organ Component)	2
C0333343	Body cavities	biologic_function	anatomy (T030 Body Space or Junction)	3
C0024919	Maternal Behavior	biologic_function	behavior (T054 Social Behavior)	3
C0025564	Metaphase	anatomy	biologic_function (T043 Cell Function)	2
C0019054	hemolysis	disorder	biologic_function (T043 Cell Function)	2
C0013081	Down-Regulation	procedure	biologic_function (T043 Cell Function)	2
C0086250	Erythrocyte Sedimentation Rate	gene_or_protein	biologic_function (T043 Cell Function)	2
C0039971	Thirst	disorder	biologic_function (T039 Physiologic Function)	4
C0001038	Acetylation	gene_or_protein	biologic_function (T044 Molecular Function)	3
C0376154	Skin callus	biologic_function	disorder (T020 Acquired Abnormality)	3
C0079380	Frameshift Mutation function	biologic_function	disorder (T049 Cell or Molecular Dysfunction)	4
C0332472	abnormal fragmented structure	biologic_function	disorder (T190 Anatomical Abnormality)	3
C0007778	Cerebral Decortication	disorder	procedure (T061 Therapeutic or Preventive Procedure)	2
C0451022	Beck depression inventory	behavior	procedure (T060 Diagnostic Procedure)	5
C0079319	Evoked Potentials, Auditory, Brain Stem	biologic_function	procedure (T060 Diagnostic Procedure)	2
C0178695	hyaluronate	microorganism	substance (T118 Carbohydrate) (T123 Biologically Active Substance) (T121 Pharmacologic Substance)	2
C0034153	Products Used to Treat Thrombocytopenic Purpura	disorder	substance (T121 Pharmacologic Substance)	6

*The “GS class ranking” means how low (possibly 2~7) the supposed correct class was put down in the ranking of similarity scores by the distributional classifier. For example, 5 means the gold standard class was considered to be the 5th possible class which the CUI should belong to.
