# Supplementary material for: Using contextual and lexical features to restructure and validate the classification of biomedical concepts
Source: BMC Bioinformatics. 2007 Jul 24;8:264. doi: 10.1186/1471-2105-8-264 (PMC2014782; doi:10.1186/1471-2105-8-264)
Supplement: Additional file 3 — Summary of the 22.5 misclassifications by the string-based approach without parenthesized annotations. A more detailed list of the misclassified concepts by the string-based approach without using the parenthesized annotations. [file 1471-2105-8-264-S3.pdf]

Summary of the 22.5 misclassifications by the string-based approach without parenthesized annotations

CUI	Concept	Predicted	Gold Standard (GS) class & SN types	GS class ranking*
C0442037	Popliteal	procedure	anatomy (T029 Body Location or Region)	3
C0225328	Fibril	disorder	anatomy (T026 Cell Component)	3
C0431085	[M]Unspecified tumor cell NOS	disorder	anatomy (T025 Cell)	3
C0037004	Shoulder	disorder	anatomy (T023 Body Part, Organ, or Organ Component)	2
C0694756	Intrauterine	procedure	anatomy (T030 Body Space or Junction)	5
C0442034	peritoneal	procedure	anatomy (T029 Body Location or Region)	4
C0016504	Foot	disorder	anatomy (T023 Body Part, Organ, or Organ Component)	2
C0162388	Killing	biologic_function	behavior (T054 Social Behavior)	6
C0019054	hemolysis	procedure	biologic_function (T043 Cell Function)	3
C0039971	Thirst	disorder	biologic_function (T039 Physiologic Function)	4
C0086250	Erythrocyte Sedimentation Rate	procedure	biologic_function (T043 Cell Function)	2
C0015895	Fertility	disorder	biologic_function (T040 Organism Function)	3
C0221117	Anergy	procedure	biologic_function (T043 Cell Function)	2
C0079380	Frameshift Mutation function	biologic_function	disorder (T049 Cell or Molecular Dysfunction)	4
C0085281	Addictive Behavior	behavior	disorder (T048 Mental or Behavioral Dysfunction)	2
C0071728	porin	substance	|gene_or_protein (T116 Amino Acid, Peptide, or Protein)	3
C0451022	Beck depression inventory	disorder	procedure (T060 Diagnostic Procedure)	2
C0042210	Vaccines	disorder	substance (T121 Pharmacologic Substance)	2
C0282563	Macrolides	disorder	substance (T109 Organic Chemical)	2
C0034428	Quinolones	disorder	substance (T109 Organic Chemical)	2

C0002073	Alkylating Agents	disorder	substance (T131 Hazardous or Poisonous Substance) (T121 Pharmacologic Substance)	2
C0387678 (0.5)	frataxin	disorder	substance (T123 Biologically Active Substance)	3
C0034153	Products Used to Treat Thrombocytopenic Purpura	disorder	substance (T121 Pharmacologic Substance)	3

*The "GS class ranking" means how low (possibly 2~7) the supposed correct class was put down in the ranking of similarity scores by the distributional classifier. For example, 5 means the gold standard class was considered to be the 5th possible class which the CUI should belong to.
